# Three-Dimensional Bioprinting: The Ultimate Pinnacle of Tissue Engineering

**DOI:** 10.7759/cureus.58029

**Published:** 2024-04-11

**Authors:** Parkavi Arumugam, G Kaarthikeyan, Rajalakshmanan Eswaramoorthy

**Affiliations:** 1 Periodontics, Saveetha Dental College and Hospitals, Saveetha Institute of Medical and Technical Sciences, Saveetha University, Chennai, IND; 2 Centre of Molecular Medicine and Diagnostics (COMManD), Department of Biochemistry, Saveetha Dental College and Hospitals, Saveetha Institute of Medical and Technical Sciences, Saveetha University, Chennai, IND

**Keywords:** tissue regeneration, stem cells, growth factors, extrusion-based bioprinting, bioink, three-dimensional bioprinting

## Abstract

Three-dimensional (3D) bioprinting has emerged as a revolutionary additive manufacturing technology that can potentially enable life-changing medical treatments in regenerative medicine. It applies the principles of tissue engineering for the printing of tissues and organs in a layer-by-layer manner. This review focuses on the various 3D bioprinting technologies currently available, the different biomaterials, cells, and growth factors that can be utilized to develop tissue-specific bioinks, the different venues for applying these technologies, and the challenges this technology faces.

## Introduction and background

Regenerative medicine is an upcoming domain of medical science that is focused on replacing the tissues lost due to trauma and disease processes. It employs tissue engineering principles to regenerate the tissues to restore form, function, and esthetics. With many affected individuals worldwide requiring the replacement of lost tissues, developing a personalized, predictive therapeutic option is the need of the hour. Three-dimensional (3D) bioprinting is a form of tissue engineering approach using additive manufacturing that employs 3D imaging modalities and computer-aided design software to three-dimensionally bioprint tissues and organs layer by layer using a plethora of biomaterials in a customized and specific pattern [1]. This technology's versatility, customization, and preciseness provide it an edge over other conventional scaffold-based regenerative modalities, which cannot mimic the complex structural, biological, and spatial distribution of complex tissues [2]. It has the potential for bioprinting tissues and organs, reducing the exponential demand for organ transplants. It would also enable the bioprinting of in vitro tissue models for pharmaceutical analysis, reducing the need for animal model testing. Moreover, 3D printing technologies using additive manufacturing are more environment-friendly than subtractive manufacturing and conventional manufacturing. The use of predominantly natural components for bioprinting applications makes them more biocompatible, biodegradable, and environment friendly. It has gained widespread use in pharmaceuticals and the healthcare industry. With the advances in biomedical and tissue engineering approaches, 3D bioprinting has emerged as a potential panacea, making 3D bioprinted tissues and organs a reality. We are now at the precipice of a new manufacturing era with four-dimensional (4D) printing, which also considers the 4th dimension of time.

Evolution of 3D bioprinting technology

3D printing technology was first developed by Charles Hull in the year 1984 with the introduction of stereolithography (SLA). Over the years various types of 3D printing technologies have been developed and explored such that bioprinting of tissues and organs is now a reality. Table 1 outlines the evolutionary process of 3D bioprinting technology till its current state.

**Table 1:** Evolution of 3D bioprinting SLA: stereolithography; SLS: selective laser sintering. Table credits: Parkavi Arumugam.

Year	Developments in the field of 3D printing
1984	Charles Hull developed the first 3D printer - SLA
1986	SLS was invented
1988	Klebe demonstrated cytoscribing technology
1999	1st organ (urinary bladder) was printed using 3D printing at Wake Forest Institute
2000	1st extrusion-based 3D bioplotter was developed
2003	1st inkjet-based 3D printer was developed
2009	1st 3D bioprinted skin by Lee & co
2013	Emergence of 4D printing
2015	Cellink introduced 1st commercial bioink
2019	1st 3D functional heart bioprinted at Tel Aviv University
2024	5D bioprinting

The 3D bioprinting process

The process of 3D printing follows the same core principles that can be adapted for the bioprinting process, too. It consists of three stages, namely, the pre-processing stage, the processing stage, and the post-processing stage [3]. Table 2 depicts the various stages of bioprinting.

**Table 2:** The different stages of the 3D bioprinting process STL: standard triangle language. Table credits: Parkavi Arumugam.

Stages of 3D bioprinting	Procedure
Pre-processing stage	Acquisition of 3D images
	Conversion of 3D images to STL format for computer-aided designing
	Selection of 3D bioprinting technology
	Development of tissue-specific bioink
Processing stage	3D bioprinting of the constructs
Post-processing stage	Tissue maturation
	In vivo grafting of 3D bioprinted constructs

Pre-processing Stage

This is the first stage, which involves the 3D imaging of the object to be printed. These images can be obtained through 3D image software, cone beam computed tomography, computed tomography, magnetic resonance imaging, etc. [4]. The images are sliced and converted to standard triangle language (STL) files using computer-aided design (CAD) software. The most used 3D printing software are Fusion 360 (Autodesk, San Francisco, CA), TinkerCAD (Autodesk), Onshape (PTC, Boston, MA), and SolidWorks (SolidWorks Corp., Waltham, MA). The specifications, dimensions, and pattern of the object to be printed are designed and fed into the software. The selection of the 3D printing technology, the biomaterial of choice, and the printing parameters like nozzle size, printing speed, temperature, and pressure are decided at this stage. The formulation of the bioink lies at the core of the bioprinting process. The cells of interest, various combinations of polymeric hydrogels, and crosslinking agents constitute the bioink. The crosslinking mechanism can be initiated through the use of chemicals or light. The biomaterials of choice would dictate the quality and integrity of the tissues to be printed, affecting the regenerative outcomes.

Processing Stage

This stage involves the actual printing of the object. The formulated bioink, along with the incorporated cells, are fed into the bioprinter. According to the previously set parameters, the object is printed layer by layer. A layer of the bioink extrusion undergoes a phase change from liquid to solid upon exposure to the crosslinking agent [5]. The bioink transforms into a solid drop or filament that fuses to the adjacent layer to form the solid object. Various crosslinking agents are added to the bioink that initiates the process following exposure to light or chemicals [6].

Post-processing Stage

This stage involves the post-printing maturation of the printed object. The sacrificial support structures used during the printing stage are removed, and the bioprinted scaffolds are allowed for tissue maturation in a bioreactor with appropriate growth media under sterile conditions. Bioreactors function as simulators with a controlled environment of temperature, pressure, pH, and oxygen saturation, providing mechanical and biological cues for tissue maturation and development [7].

## Review

3D bioprinting technologies

Currently, inkjet-based bioprinting, extrusion-based bioprinting, and laser-assisted bioprinting are the 3D printing technologies conducive to tissue printing. No single bioprinting technology has yet been able to print tissue or organs at all scales and complexities [8]. Each bioprinting technology has advantages and limitations associated with the type of bioink suitable for the particular technology, as listed in Table 3 [7]. The bioink characteristics like viscosity, rheology, thixotropy, shear stress, and surface tension dictate the material's applicability [9].

**Table 3:** Comparison of the different 3D bioprinting technologies The table is obtained from Zhang et al. [7] and published under Creative Commons Attribution License 4.0.

3D bioprinting technology	Inkjet-based 3D bioprinting	Extrusion-based 3D bioprinting	Laser-assisted 3D bioprinting
Mechanism of action	Thermal or piezoelectric actuators create pressure in the bioink reservoir, resulting in drop-wise ejection of bioink	Mechanical pneumatic pressure causes the extrusion of bioink as filaments	Bioink loaded laser sensitive ribbons on exposure to laser beam cause expulsion of bioink droplets
Bioink viscosity	3.5–12 mPa/s	30 to >6 × 10^7^ mPa/s	1–300 mPa/s
Cell viability	>85%	40–80%	>95%
Printing resolution	10–100 µm	100 µm–100 mm range	~75 µm
Print speed	Fast	Slow	Medium
Cost	Low	Moderate	High
Advantages	High-speed printing, commonly available, cost-effective	Allows bioprinting with viscous inks, allows greater cell density	Highly precise
Disadvantages	Low precision	High shear stress on cells during extrusion	Technique-sensitive, the laser may be detrimental to cells, poor directionality of drops, costly

Inkjet-Based 3D Bioprinting

Inkjet-based bioprinting, also known as drop-on-demand bioprinting, was the first bioprinting technology to evolve from commercial two-dimensional (2D) inkjet printers. It was developed by replacing the cartridges of inkjet printers with bioink. It uses thermal or piezoelectric mechanisms to create pressure in the bioink reservoir that results in the expulsion of droplets of the bioink from the nozzle [8]. These droplets, printed in a layer-by-layer pattern, fuse to form a solid structure post-cross-linking. Thermal inkjet printers employ electric heating of the printer head to create pressure, resulting in droplet ejection [10]. Studies have shown that thermal heating increases the bioink temperature to 200-300°C. However, there is no significant effect on the cell viability and the cellular deoxyribonucleic acid (DNA) characteristics, implying its biocompatibility with the incorporated cells [11]. It has been shown that short-duration heating increases the bioink temperature by 4-10°C only [12]. However, nozzle clogging, increased temperature to cells, nonuniform droplet size, and directionality are some of the disadvantages associated with thermal printers. In piezoelectric printers, when voltage is applied across the piezoelectric material, it causes a change in the piezoelectric crystal structure, causing acoustic waves that create pressure in the bioink, leading to the ejection of droplets of bioink at the nozzle [13]. It can control the droplet size and directionality by adjusting the pressure in the printer head. It also avoids the exposure of the cells to heat.

The limitation associated with inkjet printers is the need to use a bioink of low viscosity with low cell concentrations. Highly viscous liquid bioinks require greater pressure for droplet formation, leading to increased shear stress on the cells during ejection from the nozzle. Also, high bioink cell concentration has been associated with poor crosslinking mechanisms, increased nozzle clogging, and increased shear stress at the nozzle tip [14]. Even with these limitations, inkjet printers have several advantages: high printing speed, better printing resolution, low cost, multi-material printing, and compatibility with biomaterials. Inkjet printing has been applied in the bio-fabrication of skin and cartilage tissues.

Extrusion-Based 3D Bioprinting

Extrusion-based bioprinting is the most used and the go-to technology for bioprinting of tissues. Here, the bioink is extruded as strands or filaments instead of droplets. These strands, deposited layer by layer, fuse together to form the final tissue construct. Mechanical and pneumatic mechanisms are employed to extrude the bioink through the nozzle [15]. The mechanical method uses a screw or piston for material extrusion and has been shown to have greater control and spatial resolution over the amount of extruded biomaterial [16]. The pneumatic method uses air pressure for material extrusion and hence has poorer control over the extrusion of low-viscosity bioinks [10]. One of the main advantages of extrusion-based bioprinting is the ability to print high-viscosity bioinks with high cell density. Adequate cell density is often required for the biofabrication of physiologic tissue scaffolds, which these printers favor. However, highly viscous bioinks require higher extrusion pressure, which causes excessive shear stress on the cells while extrusion, affecting their viability [17]. Lower cell viability is the major disadvantage of microextrusion printers. Studies have shown a cell survival rate of 40-86% with microextrusion printers, which is lower than that observed with inkjet printers [18]. Achieving the optimum viscosity with high cell density, allowing for good print resolution and cell viability, should be the ideal strategy for extrusion-based bioprinting applications. Extrusion-based technology has been applied for bioprinting various biological systems and tissue scaffolds like bone, skin, cartilage, aorta, cardiac and muscle tissue, etc.

Laser-Assisted 3D Bioprinting

Laser-assisted bioprinting works on the principle of laser-induced forward transfer. Here, donor material is in the form of a ribbon with three layers [10]. The topmost layer is transparent, usually glass, allowing for laser transfer. The middle layer is a laser-absorbent layer of metallic substances like gold and titanium. The bottom layer is made of bioink material. The middle layer absorbs the laser beam propelling a high-pressure droplet of being on exposure of the ribbon to the laser beam. The laser parameters like the wavelength, intensity, pulse duration, and viscosity of the bioink determine the resolution of the printed constructs. It is a nozzle-free and non-contact method of bioprinting, allowing for high cell density and cell viability. It is also compatible with various biomaterials with different viscosities. However, preparing donor ribbon strips with complex bio-inks is a cumbersome process. Residual metallic contamination of the bioink from the ribbon layers, poor directionality of droplet deposition, and high cost are the limitations of these printers [4].

Bioinks for 3D bioprinting

Bioinks are the amalgamation of biomaterials with cells and growth factors of interest used for bioprinting tissues. They are hydrogel-based materials maintained in a fluid state with appropriate rheological parameters conducive to bioprinting. The bioinks contain crosslinking agents, which, on activation post extrusion, cause the curing of the bioink to solid structures. Three types of crosslinking mechanisms are currently being used: physical, chemical, and thermal crosslinking methods [6]. Physical crosslinking includes the use of light and ionic bonds. Chemical crosslinking includes using enzymatic agents and chemical substances while thermal crosslinking includes using heat.

Bioinks are the crucial components of the bioprinting process that determines the nature of the tissue constructs printed. The viscosity, thixotropic, pH, temperature, concentration, shear stress, surface tension, printability, stacking ability, cell density, biocompatibility, non-immunogenicity, biodegradability, swelling ratio, cell adhesion, and cell proliferation are significant parameters that determine the applicability of a biomaterial as a bioink [19]. Moreover, the cells incorporated in the bioink and the tissues that interact with the 3D bioprinted constructs also dictate the outcome, with different cell types reacting differently to the same bioinks. An ideal bioink should possess adequate viscosity, shear thinning, and gelation ability that enables extrusion of the biomaterial as a strand or drop through the nozzle that solidifies post-curing while providing adequate mechanical strength to maintain the shape fidelity and resolution of the scaffold post-printing. In regards to the biological properties, the bioink should exhibit biocompatibility, biomimeticity, and biodegradability, enabling adequate cell viability, cell adhesion, cell migration, and cell proliferation [20].

Various hydrogel biomaterials have been employed as the base of the bioink, which holds the other components of the bioink, like the cells, growth factors, crosslinking agents, and fillers. They can be classified into biomaterials (as depicted in Table 4) derived from natural sources, synthetic sources, composite biomaterials, and commercial bioinks. Natural origin biomaterials can be further classified into protein-based, polysaccharide-based, and decellularized extracellular matrix (DECM)-based biomaterials based on the nature of the material. The various bioink components that are used for bioprinting applications exhibit the property of biodegradation and hence are more biocompatible. Post-implantation in the body, these bioprinted scaffolds undergo a remodeling process through which new tissue deposition occurs leading to the incorporation of the scaffolds into the native defect site. During this process, the bioprinted scaffold undergoes biodegradation paving the way for new tissue formation. The challenge lies in achieving a balance between the rate of degradation of the bioprinted scaffold and the rate of deposition of new tissue. It is specific to each tissue type and hence, the appropriate selection of the bioink components is highly crucial for the successful integration of the bioprinted scaffold for enhanced regeneration. Though the commonly used natural and synthetic components have been shown to degrade into non-toxic residues, further studies need to be conducted to assess the long-term effects of these bioprinted scaffolds on the body.

**Table 4:** The various hydrogel biomaterials used for bioprinting DECM: decellularized extracellular matrix. Table credits: Parkavi Arumugam.

Natural biomaterials	Synthetic biomaterials	Composite biomaterials	Commercial biomaterials
Collagen, gelatin, fibroin-silk, agar, hyaluronic acid, dextran, gellan gum, allogenic DECM-based gel, matrigel	Polyethylene glycol, polycaprolactone, pluronic	Bioink that is a combination of natural and synthetic components	Cellink, DermaMatrix, Novogel

Protein-Based Biomaterials

Collagen: Collagen is a ubiquitous protein of the mammalian extracellular matrix that plays a significant role in wound healing and tissue regeneration. It is a highly biocompatible material that provides biomimetic cues for cell differentiation and proliferation. It retains a structural and functional similarity to the native tissues, enabling the creation of a microenvironment that orchestrates the events toward the regeneration of the tissues [21]. Collagen can be obtained from various animal and marine sources. It is hydrophilic, with good cell affinity, adhesive, and proliferative properties. However, it has poor mechanical strength and an unpredictable degradation rate that can be modified by adding synthetic polymers. A recent systematic review of collagen bioinks has concluded that pure collagen bioinks with additives produce sufficiently strong scaffolds. However, they are associated with poor cell viability and proliferation. Thus, the current research focuses on improving the biomimetic effect of high-concentration collagen bioinks [22].

Gelatin: Gelatin is a denatured form of collagen that has been explored as a bioink. It is a biocompatible and biodegradable thermo-sensitive hydrogel with good gelation properties, low immunogenicity, and high cellular affinity. It has the presence of intrinsic arginyl-glycyl-aspartic acid (RGD) motifs that retain the functional properties of the protein. This protein's main advantage is its wide crosslinking ability, ranging from thermal to physical or chemical. It has better mechanical properties compared to collagen and hence is often used in combination with collagen for improved bioink properties. YP Singh et al. developed crosslinker-free bioink using gelatin, silk, and chondrocytes and printed 3D cartilaginous tissue constructs that showed good mechanical properties, printing fidelity, and cell proliferation with extracellular matrix (ECM) deposition [23]. Another group of researchers developed an all-gelatin-based toolbox with human dermal microvascular endothelial cells and human adipose-derived stem cells that was used for printing vasculogenic components cocultured with osteogenic components. The bioprinted constructs revealed the vascular structure formation, which positively affected the bone matrix deposition [24].

Silk-fibroin: Silk-fibroin obtained from *Bombyx mori* (*B. mori*) or silkworms have also gained popularity due to their good mechanical strength, elasticity, low immunogenicity, biocompatibility, biodegradability, and cell encapsulation properties. Their rheological properties and degradation rate have been a cause of concern for which combinations of synthetic polymers can be added. Sharma et al. (2019) developed a calcium-conjugated silk fibroin-gelatin bioink for bone bioprinting applications. They concluded that the bioink showed the enhanced osteogenic potential of human mesenchymal stem cells by upregulating various gene expressions such as Runt-related transcription factor 2 (RUNX2), collagen type 1 (COL1), alkaline phosphatase (ALP), bone morphogenetic protein 2 (BMP-2), and bone morphogenetic protein 4 (BMP-4) [25].

Polysaccharide-Based Biomaterials

Alginate: Alginate is a natural complex polysaccharide obtained from brown seaweed. It has β-D-mannonic acid and α-L-guluronic acids linked through 1,4-glycosidic bonds. It has high structural similarity with glycosaminoglycans of the ECM. It has excellent biocompatibility and biodegradability and is available at a cheaper cost. It has wide applicability in the dental, food, and pharmaceutical industries. It has good gelation ability when exposed to calcium ions and induces less shear stress on the cells during extrusion. However, it is a bioinert material, and higher alginate concentrations have been associated, with poor cell viability and cell adhesiveness. Christensen et al. (2015) applied mouse fibroblast-based alginate bioinks to print vascular-like cellular structures with horizontal and vertical bifurcations using liquid-supported inkjet printing [26]. Daly et al., in their proof of principle approach, showed that it is possible to create in vitro hypertrophic cartilage templates by employing stem cells in a gamma-irradiated alginate bioink that includes adhesion peptides like Arg-Gly-Asp [27].

Hyaluronic acid: Hyaluronic acid is an anionic, non-sulfated glycosaminoglycan abundantly present in cartilage, bone, and skin tissues. It is a disaccharide polymer D-glucuronic acid and N-acetyl-D-glucosamine, linked via alternating β-(1→4) and β-(1→3) glycosidic bonds. It plays a key role in wound healing, tissue regeneration, and resolution of inflammation. It is highly hydrophilic with enhanced lubricating function. The US FDA has approved it for use as dermal fillers [28], intra-articular injections, beauty products, etc.

Dextran: It is a biodegradable complex bacterial exopolysaccharide that is made up of repeating glucose units. It is biocompatible, hydrophilic, and exerts anti-thrombotic, anti-inflammatory properties. It has good viscoelastic and rheologic properties and has been used in food, pharmacology, and tissue engineering.

Chitosan: Chitosan is derived from chitin, obtained from crustaceans' exoskeleton. It has been proven to possess antimicrobial, hemostatic, and wound-healing properties. Studies have explored its potential in the bioprinting arena and stated that it can be combined with synthetic polymers that improve its mechanical and handling characteristics [29].

Agar: Agar is another thermo-responsive, biocompatible, and biodegradable polysaccharide obtained from marine sources. It is commonly used in the food, pharmaceutical, and dental industries. It has excellent gelation and rheological properties conducive to bioprinting applications. Studies have shown that bioinks with agarose have expressed greater cell viability [30].

DECM-Based Biomaterials

DECM-based bioinks have recently gained significant attention due to their structural and functional similarity to the native ECM. They are allogenic and xenogenic biomaterials that, when exposed to physical, chemical, and/or enzymatic decellularization protocols, lead to removing all immunologic components like cells and nuclei while retaining the extracellular matrix and protein. This ECM provides a biomimetic effect concerning the structural composition and biological signaling mechanism. The DECM can be extracted to formulate bioinks that can be used for bioprinting. Reseeding the bioprinted scaffolds with appropriate cells creates a microenvironment that is similar to the native tissues, enabling cell differentiation and proliferation. Pati et al. proved the versatility of the bioprinting process by utilizing tissue-specific DECM bioinks of adipose, cartilage, and cardiac tissue to print scaffolds with biomimetic functions [31].

Synthetic Polymer-Based Biomaterials

Polyethylene glycol: Polyethylene glycol (PEG) is a synthetic polymer commonly used for bioprinting due to its good mechanical strength, shape fidelity, biocompatibility, and non-immunogenicity. Though it is a bioinert material that does not degrade on its own, its chemical nature gives vast scope for tailoring its behavior by modifying its chain length and adding natural hydrogels. Molecularly engineered PEG and gelatin-based bioink have also shown optimum rheological properties for extrusion with improved shape fidelity printing accuracy while maintaining cell viability.

Polycaprolactone: Polycaprolactone (PCL) is yet another thermoplastic polymer commonly used for bioprinting for bone and cartilage tissue. It is a biocompatible and biodegradable polymer that the Food and Drug Administration (FDA) has approved for applications in the medical field. It has good mechanical strength. However, it has a few disadvantages, such as poor cell affinity and hydrophobicity [32]. Inorganic fillers have been added to it to increase its functionalities and improve its mechanical properties.

Pluronic: Pluronic F-127 is a sacrificial bioink used to print and support structures [33]. It is a thermosensitive poloxamer that has good printability and viscoelasticity. It has surfactant properties with both hydrophilic and hydrophobic ends that can used for modification of its properties. It lacks the biological properties for cell adhesion and long-term cell survival. It has been commonly used for bioprinting vascular structures and organs on chip-model systems.

Commercial Biomaterials

Bioprinting inducting has developed over the years, emerging with patented bioink formulations that are commercially available. CELLINK (Sweden, Gothenburg), the leading industry specialist, has a range of ready-to-use, tissue-specific, and photo-ink combinations of collagen, gelatin, PEG, chitosan, pluronic, etc. TissueFab and Novogel are other examples of commercially available bio-inks currently used in bioprinting applications.

Cells for bioprinting

Tissue regeneration is a spatiotemporal complex and well-orchestrated process requiring an appropriate cell selection. Obtaining well-characterized cells from reproducible sources has been an important issue in bioprinting. Cell sources considered for bioprinting are either stem cell origin or functional tissue-specific cells. Stem cells express the properties of self-renewal and differentiation. Adding stem cells to the bioink greatly reduces the complexity of bioprinting by minimizing the different cell types to be included. However, it requires the maintenance of specific culture media conditions to differentiate the stem cells into the appropriate cell phenotypes of interest. This would also require the addition of specific growth factors and signaling molecules. Stem cells from various origins like pulp and periodontal ligament-derived stem cells, adipose tissue-derived stem cells, human amniotic fluid-derived stem cells, hematopoietic stem cells, and mesenchymal stem cells have been currently employed for bioprinting applications [34]. In contrast, functional-specific cells provide greater control over directing the regenerating outcomes. Osteoblasts, chondrocytes, cartilage and muscle tissue cells, and epithelial cells are a few of the cells of interest used in bioprinting applications.

Growth factors for bioprinting

When added to the bioink, growth factors, and signaling molecules, provide direction for appropriate cell differentiation and proliferation [35]. Growth factors are crucial in embryonic development, tissue morphogenesis, cell proliferation, and cell differentiation. Bone morphogenetic proteins (BMP), platelet-derived growth factors (PDGF), and transforming growth factors (TGF) have been applied for bone tissue engineering. In contrast, vascular endothelial growth factor (VEGF), fibroblast growth factor (FGF), hepatocyte growth factor (HGF), and insulin-like growth factor (IGF) have been applied for angiogenesis, wound healing, and vascular printing. Factors such as the concentration, bioavailability, stability, pH, delivery route, diffusion, release kinetics, and host response all dictate the efficacy of growth factor delivery. Hydrogels, capsules, liposomes, microspheres, fibers, and nanotubes are the commonly used delivery methods for growth factor delivery.

Post-printing scaffold maturation

The bioprinting scaffolds must be cultivated in culture media to allow scaffold maturation before in vivo placement. Static and dynamic incubators that contain culture media are used to supply oxygen and nutrients and exchange waste products [7]. Static bioreactors allow scaffold maturation in a static environment with the need for manual renewal of culture media. In contrast, dynamic bioreactors allow for the maintenance of the pH, oxygen saturation, temperature, and mechanical stimuli. Mechanical stimuli allow for greater proliferation and differentiation of the cell, thus improving the maturation of the bioprinted tissue construct. Spinner flasks, rotating wall vessels, compression, and perfusion bioreactors are examples of dynamic bioreactors used for scaffold maturation [7].

Bioprinting applications

Bone and Cartilage Bioprinting

Bone bioprinting has become integral to regenerative medicine for surgical reconstruction of bony defects following trauma, tumor resection, and bone diseases. Customized 3D metal scaffolds have been printed using titanium-based alloys to reconstruct bony defects. 3D scaffolds have also been printed for efficient surgical planning of complicated cases to guide surgeons and trainee doctors in effective management. It gives an idea about the different tissue planes and vasculature during surgery. Extending this knowledge to improve personalized care further, 3D bioprinted bone scaffolds have become a reality. Various bone bioink formulations using predominantly gelatin methacryloyl (GelMA), alginate, polyethylene glycol (PEG), and bone substitutes like hydroxyapatite, bioglass, and biphasic calcium phosphates have been developed. They are then bioprinted using extrusion-based technology into osseous scaffolds that are patterned according to the defect dimension obtained through CAD designing. This also enables overcoming the issues associated with conventionally used bone grafting, such as donor site morbidity, poor tissue integration, and infectious transfer. Various bone bioinks have been formulated and explored to achieve a structure with mechanical and biological properties similar to living bone [36].

Vascular Bioprinting

Incorporating 3D bioprinted vascular channels has been a significant challenge in bioprinting. Coaxial bioprinting with multiple bioinks has been employed for vascular printing. Sacrificial bioinks like pluronic have emerged as the material of choice for printing vascular structures [37]. Post crosslinking, the scaffolds are exposed to appropriate temperature gradients to ensure complete removal of the thermosensitive pluronic hydrogel. Manual removal of the sacrificial material through aspiration has also been tried, creating hollow tubular structures that are cultured to form vasculatures.

Neuronal Bioprinting

Printing tissue scaffolds with the same function and feel has been the ultimate goal of bioprinting technologies. Achieving innervation through the incorporation of nerve cells has been ambiguous. Conduits of neuronal tissues have been printed with Schwann cells and glial cells with greater resolution than previously reported methods [38].

Skin Bioprinting

Skin bioprinting is another tissue of need for treating burn patients following trauma, infections, cancer, etc. Skin bioprinting has been performed by incorporating epithelial and fibroblast cell types [39]. Sweat glands have also been included in these scaffolds. The dermis and epidermis layers have been printed and attached to animal skins.

Drug Screening and Organ-on-a-Chip

Bioprinting has enabled drug screening with uniformly seeded cells with good cell densities on microdevices. Organ-on-a-chip models have been developed to provide organ models with typical functionalities to assess the interaction of newer drugs with the tissues. It reduces the use of animal models for preliminary investigations to a greater extent, thereby making pharmaceutical drug screening studies comparatively safer for animals than before [40]. Table 5 compares the various parameters used for tissue-specific bioprinting applications [7].

**Table 5:** Comparison of tissue-specific 3D bioprinting PEG: polyethylene glycol; PLGA: poly lactic-co-glycolic acid; PCL: polycaprolactone; hMSC: human mesenchymal stem cells. The table is obtained from Zhang et al. [7] and published under Creative Commons Attribution License 4.0.

Tissue	Bone printing	Skin printing	Cardiac tissue printing	Vascular printing
Bioinks	PEG, PLGA, gelatin, alginate, collagen, chitosan, bioglass	Collagen, gelatin-alginate, PEG, fibrin	PEG, PCL, fibrin, alginate, gelatin	Hyaluronic acid, fibrin, pluronic, PLGA, PEG
Cells	Human mesenchymal stem cells (hMSC)	Human dermal fibroblasts, human keratinocytes	Bone marrow-derived hMSC, human cardiac progenitor cells, human umbilical vein endothelial cells	Human mesenchymal stem cells, adipose stem cells
Bioprinting technology	Extrusion	Extrusion, laser-assisted	Extrusion, inkjet	Extrusion

Challenges and future prospects

Bioprinting is still in its infancy and bogged down by various challenges related to different aspects of the bioprinting process. The primary challenge is developing an ideal bioink that is apt for the tissue of interest to be printed. Maintaining adequate cell density and viability following extrusion, obtaining air bubble-free extruded filaments, achieving adequate mechanical strength post-printing, achieving vascularization and innervation of the tissue constructs, and printing complete organs are the major challenges slowing down the bioprinting process. Research is being conducted to overcome these hurdles and provide personalized treatment solutions for regenerating the lost tissues. Gaining in-depth knowledge regarding the organogenesis process, the tissue structure, composition, and behavior of each tissue, ways to maintain cell viability, and tissue integration with the native tissue post-printing would enable us to overcome these challenges one step at a time. 4D bioprinting has recently emerged, where time is considered the fourth dimension of printing. The printed scaffold modulates their organization and behavior according to time-dependent external stimuli. The future is moving toward five-dimensional (5D) printing, which will occur in multiple rotational axes. Advanced bioprinting technologies along with the integration of artificial intelligence would greatly reduce the demand for organ donations. Government organizations and regulators are still working toward achieving a balance between the need for organ donation through early prevention and management of diseases and improved procurement of organs. Application of bioprinting technologies would greatly reduce the burden on the governments and buy us time till every nation becomes self-sufficient to manage the need for organ donations. Though the setting up of a bioprinting center of excellence is a costly affair, in terms of obtaining the infrastructural and biologics support, the number of lives saved through its applications in regenerative and rehabilitative medicine is of paramount significance.

## Conclusions

The advancement of 3D bioprinting holds extraordinary promise in transforming regenerative medicine, offering a groundbreaking avenue to diminish reliance on organ transplants and animal experimentation. There exists a pressing need for continued exploration to refine the ideal bioink, enabling customization for diverse tissue-specific bioprinting endeavors. This pursuit promises to usher in a new era of personalized and predictive treatment methodologies, tailoring interventions to the unique needs of individual patients. With further research and development, the potential applications of 3D bioprinting can extend far beyond current limitations, offering hope for more effective and efficient medical interventions. Such advancements have the potential to redefine the landscape of health care, offering tailored solutions and mitigating the challenges associated with organ scarcity and ethical concerns surrounding animal testing. It is imperative to harness this technology's capabilities to their fullest extent, driving innovation and paving the way for a brighter future in regenerative medicine.
